# Spatial distribution of HIV/AIDS risk in Campinas-SP (1980–2019): a geospatial analysis by decade

**DOI:** 10.1590/1980-549720260021

**Published:** 2026-05-08

**Authors:** Márcio Cristiano de Melo, Celso Stephan, Ricardo Carlos Cordeiro, Valeria Correia de Almeida, Maria Rita Donalisio

**Affiliations:** I Faculdade de Medicina São Leopoldo Mandic – Araras (SP), Brazil.; II Universidade Estadual de Campinas, Department of Collective Health – Campinas (SP), Brazil.; III Secretaria Municipal de Saúde de Campinas, Department of Health Surveillance – Campinas (SP), Brazil.

**Keywords:** HIV, AIDS, Spatial analysis, Public health surveillance, Socioeconomic factors, Epidemiology

## Abstract

**Objective:**

We aimed to analyze the spatial distribution of AIDS case risk (1980–2019) and HIV infection risk (2000–2019) in Campinas, Brazil.

**Methods:**

We conducted a geospatial case-control study using the Notifiable Diseases Information System data. We included all reported AIDS and HIV cases among individuals aged 13 years or older, excluding pregnant women and death notifications. We geocoded the data using the official cartographic base of the municipality and UTM coordinates (SAD69). We modeled spatial risk using generalized additive models (GAMs), applying the Kelsall and Diggle method with pairs of geographic coordinates as non-parametric predictors. We stratified the results by decade and compared them across periods.

**Results:**

We identified 11,372 AIDS cases (1980–2019) and 3,329 HIV cases (2000–2019). We observed a persistent pattern of high risk in the central district (Distrito Sede) since the 1980s, with progressive expansion toward peripheral districts (Campo Grande, Ouro Verde, Sousas, and Joaquim Egídio). This peripheralization of the epidemic coincided with accelerated urbanization and socio-spatial inequality. The highest incidence rates of HIV and AIDS occurred among adults aged 20 to 39 years, with a notable increase among older adults. The analysis revealed partial overlaps of hotspots and expansion of risk areas.

**Conclusion:**

The spatial distribution of HIV/AIDS in Campinas is heterogeneous and dynamic, characterized by persistence in central areas and expansion into vulnerable peripheral regions. Georeferencing was a strategic tool to guide territorially sensitive prevention and control actions.

## INTRODUCTION

Over time, the spread of HIV infection has affected Brazil's major metropolises, subsequently advancing into medium- and small-sized municipalities. In addition to geographic variability in access to diagnosis and treatment, a concentration of the epidemic within key populations is observed, as indicated by data from the Ministry of Health^1,2^.

A higher rate of case detection in state capitals and metropolitan centers has been noted since the onset of the epidemic^3^. These centers possess the highest population densities and exhibit greater interaction with smaller cities, taking into account the ease of travel and the presence of more extensive opportunities for employment and business, as well as a greater availability of social and urban infrastructure^4,5^.

The state of São Paulo has been characterized by a progressive increase in the detection of AIDS cases across the majority of its municipalities throughout the historical trajectory of the disease. *In situ* growth was observed, that is, growth occurring within the same locality and independently of sociodemographic variables. The risk of infection increased and spread throughout the state starting in the 2000s, thereby evidencing the process of the epidemic's expansion into the interior regions^6^.

Analyzing the spatial distribution of infection allows for the identification of areas with a relatively higher concentration of risk, thereby enabling the territorial prioritization of interventions. Thus, a spatial risk approach goes beyond merely reporting the spatial distribution of cases, as it considers notifications by taking into account the population at risk^7^.

The spatial distribution of the epidemic exhibits distinct patterns, characterized by a higher concentration of cases in neighborhoods with poorer socioeconomic conditions, areas where cases predominantly occur among men. Another observed pattern involves a higher susceptibility to infection among women in peripheral neighborhoods, where the ratio of male-to-female infections is lower. Such territorial patterns facilitate the targeting of vulnerable populations by health sector agencies^8^.

The objective of this study was to analyze the spatial distribution of the risk of AIDS cases (based on notifications) over four decades (1980 to 2017), as well as HIV cases starting from the year 2000, within the municipality of Campinas.

## METHODS

We conducted a geospatial case-control study examining the spatial risk of AIDS and HIV based on the entirety of reported cases in both categories among individuals over 13 years of age in the municipality of Campinas, SP. The cases were recorded by the Health Surveillance Department (DEVISA) of the Campinas Municipal Health Secretariat between 1980 and 2019. Following a review of the database, cases involving pregnant women were excluded because of the distinct testing protocols associated with prenatal care, which could potentially overestimate risk in areas with higher health care coverage, as were notifications where the diagnostic criterion was death where these records often lack addresses or contain imprecise location data, making geocoding unfeasible and potentially introducing spatial errors. It is acknowledged that such exclusions may result in an underestimation of risk in areas characterized by greater vulnerability.

Campinas is recognized as a hub for high-technology industrial development; it boasts a Human Development Index (HDI) of 0.805, ranking 28th among Brazilian cities. The estimated population of Campinas in 2018 was 1,194,094 inhabitants^9^. In terms of epidemiology and the control of communicable diseases, the municipality mirrors the general patterns observed in other major Brazilian cities. The city serves as a regional medical and hospital referral center; in 2017^10^, it recorded an AIDS detection rate of 17.6 cases per 100,000 inhabitants and has maintained a structured program for the prevention of and care for patients with HIV/AIDS since the onset of the epidemic.

The address variable was extracted from the epidemiological notification forms within the Information System for Notifiable Diseases (SINAN) for inclusion in the calculation of the spatial risk associated with these cases. The cartographic base developed by the Campinas Water Supply and Sanitation Society in 2000 using AutoCAD software was utilized, having been periodically updated by the Municipal Secretariat of Planning and the Municipal Secretariat of Health (with the latest update carried out in 2014). Using ArcView software, new neighborhoods, informal settlements, urban facilities, and housing complexes were incorporated, alongside updates to data relevant to the health sector. The following parameters were used in the creation of this cartographic base: the International Hayford Ellipsoid, Universal Transverse Mercator Projection, SAD69 Datum, and the municipal boundary defined by the Brazilian Institute of Geography and Statistics (IBGE) — adjusted.

The localization of cases within the cartographic base was performed using residential addresses via the geocoding method in ArcMap 10.5 software, following the correction and standardization of data against the municipality's street network database. Addresses that precluded the precise pinpointing of a location on the map were excluded — specifically those with insufficient information or inaccurate street names — as were addresses associated with the incarcerated population (because of a lack of correspondence with valid civil addresses), shelters, and long-term care facilities for people living with HIV and AIDS. Once geocoded, the cases successfully located within the database had their geographic coordinates appended in the UTM SAD69 projection system.

The method developed by Kelsall and Diggle^11^ was employed, wherein spatial risk is estimated by fitting a Generalized Additive Model (GAM), including the identification of areas exhibiting statistical significance. The selection of the Generalized Additive Model (GAM) to estimate the spatial risk of HIV/AIDS in this study is justified by its flexibility in modeling nonlinear relationships between the response variable and geographic coordinates, without the need to specify a rigid parametric structure^12^. This approach is particularly well-suited for modeling spatially continuous and smoothed risk patterns, especially when working with georeferenced point data.

For each time period, a separate model was fitted using AIDS diagnostic status and positive HIV serology as the response variable (cases = 1; controls = 0). Controls were obtained using the AmostraBrasil package—released under the General Public License (GPL)—which was developed specifically for the spatial analysis of epidemiological data^13^. The pair of geographic coordinates corresponding to the residences of cases and controls was utilized as the nonparametric predictor variable^7^. Thus, the values predicted by the model represent the estimated distribution of the risk of AIDS (1980s, 1990s, 2000s, and 2010 through 2019) and of HIV (2000s and 2010 through 2019). This period was selected because of the state-level recommendation for HIV notification issued in 2000, a measure that became nationally mandatory only in 2014^14^.

The models were computed using R software, version 3.4.1, using the GAM function from the mgcv package. The numbers on the contour lines on the map indicate the magnitude by which the risk in that region exceeds the reference value of 1, representing the average risk across the territory.

To calculate the incidence rates, the number of reported HIV and AIDS cases in Campinas, stratified by age group (13–19, 20–29, 30–39, 40–49, 50–59, and 60 years or older), was used as the numerator, while the population specific to each age group served as the denominator, covering the period between 2010 and 2019.

The project was approved by the Human Research Ethics Committee at FCM/Unicamp under No. 1.776.974.

### Data Availability Statement:

The entire dataset supporting the results of this study is available upon request to the Campinas Municipal Health Secretariat, through the Department of Education, Research, and Digital Health. The dataset is not publicly available, as it contains information that could compromise the privacy of the research participants.

## RESULTS

The analysis included 11,372 cases of AIDS diagnosed between 1980 and 2019, and 3,329 cases of HIV diagnosed during the period from 2000 to 2019. Of these, the geographic location, specifically the latitude and longitude coordinates, of 100% of the case records was used within the cartographic database.

**Figure 1:**
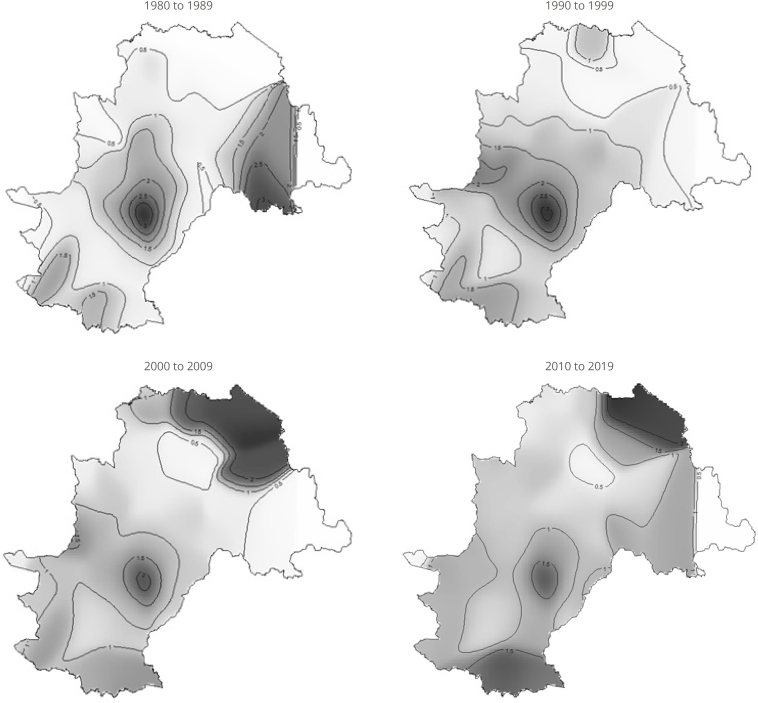
Spatial distribution of AIDS risk after generalized additive model fit across the four study periods. Campinas, SP, 1980 to 2019.

The maps in Figure 1 display the generalized linear models of the spatial distribution of AIDS risk across the four study periods. Over time, differences in AIDS risk are observed among residents within the city's territory. The color red indicates locations in Campinas where the risk of the disease was significantly higher than the average for that specific period. During the decade from 1980 to 1989, the highest risk of AIDS was evident among residents of downtown neighborhoods, in peripheral regions characterized by precarious living conditions, in the South Macro-area (specifically in neighborhoods near Viracopos Airport and adjacent to the city of Indaiatuba), and in the Joaquim Egídio District region.

In the 1990s, the highest risk continued to be observed in the Headquarters District and the South Macro-area, with an expansion into the city's North Macro-area, specifically near the Hortolândia Penitentiary Complex, located in the municipality bordering Campinas. Between 2000 and 2009, the analysis indicates a persistence of elevated risk in the municipality's Sede District and the North Macro-area (along the border with Hortolândia), while also pointing to a significant increase in risk within the Sousas District. The map presenting data for the years 2010 to 2019 indicates a higher risk of AIDS in the Joaquim Egídio District, the Sousas District, the Sede District, and the South Macro-area (Figure 1).

**Figure 2:**
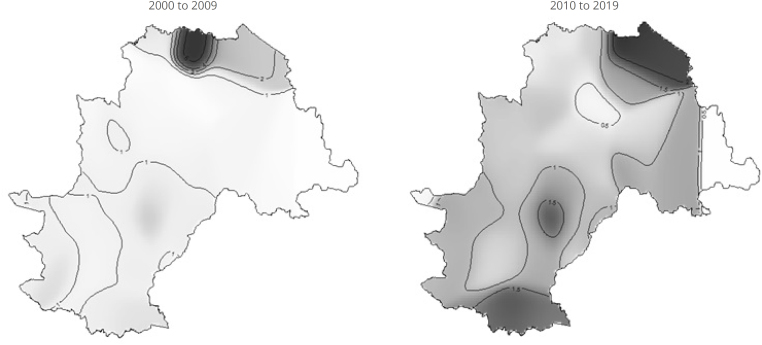
Spatial distribution of human immunodeficiency virus (HIV) risk after generalized additive model fit during the two study periods. Campinas, SP, 2000 to 2019.

Figure 2 highlights the generalized linear models regarding the spatial distribution of HIV risk between the years 2000 and 2019. During the first decade of this period (2000 to 2009), a fivefold increase in risk is evident in neighborhoods located within the North Macro-area of Campinas, specifically those belonging to the North Macro-area and the Joaquim Egídio District. In the period from 2010 to 2019, the highest risk — approximately 1.5 times higher — is observed in the East-Northeast Macro-area (Sousas District), and with the same magnitude in neighborhoods in the Sede District and the municipality's South Macro-area, bordering Indaiatuba. All the aforementioned municipalities bordering Campinas belong to the Campinas Metropolitan Region.

According to Table 1, an upward trend is observed in the rates regarding HIV cases, while a downward trend is noted for AIDS cases. The highest HIV and AIDS rates are recorded among age groups ranging from 20 to 39 years old, with figures from 2017 showing that among adults aged 20 to 29 and 30 to 39, there were 39.0 and 33.2 new HIV cases per 100,000 inhabitants, and 19.9 and 29.2 new AIDS cases per 100,000 inhabitants, respectively. A significant finding concerns the persistence of substantial HIV/AIDS rates among older adults, particularly in the age group over 60. Table 1 demonstrates that, despite having lower absolute rates, the HIV incidence rate among older adults nearly tripled between 2010 (2.2/100,000) and 2017 (5.5/100,000).

**Table 1:** Incidence rate of human immunodeficiency virus (HIV) and AIDS per 100,000 inhabitants (specific population) by age group (in years). Campinas, 2010 to 2019.

		13–19	20–29	30–39	40–49	50–59	≥ 60	Total
2010	IR HIV	1.73	35.77	32.13	17.40	13.73	2.21	19.72
	IR AIDS	0.86	37.73	55.55	45.12	23.43	11.79	32.13
2011	IR HIV	6.06	30.21	37.31	12.76	6.30	2.83	18.28
	IR AIDS	6.92	39.13	52.77	49.78	29.16	12.02	34.19
2012	IR HIV	5.18	36.72	28.16	26.56	16.17	3.39	21.34
	IR AIDS	3.45	39.23	49.55	35.42	23.10	8.13	29.20
2013	IR HIV	5.17	45.06	41.36	19.44	9.79	4.55	23.71
	IR AIDS	6.89	30.72	40.85	38.87	30.14	8.45	27.59
2014	IR HIV	5.17	58.41	43.57	18.01	19.95	3.74	27.68
	IR AIDS	3.44	29.20	35.55	34.77	19.95	3.12	22.71
2015	IR HIV	10.40	74.76	46.84	23.34	17.42	4.78	32.62
	IR AIDS	3.47	30.75	29.09	29.48	15.24	8.36	20.93
2016	IR HIV	9.63	82.08	59.15	30.82	18.59	6.30	37.86
	IR AIDS	4.38	28.97	28.84	24.78	22.88	9.16	21.01
2017	IR HIV	6.20	46.09	32.56	23.76	19.76	5.49	23.81
	IR AIDS	3.54	33.07	27.70	26.14	21.87	7.14	21.10
2018	IR HIV	13.45	115.50	82.38	43.75	30.68	7.37	52.62
	IR AIDS	0.90	23.54	22.29	21.00	11.16	4.74	15.02
2019	IR HIV	4.54	38.68	27.13	14.31	10.35	2.02	17.24
	IR AIDS	3.63	12.16	16.96	9.73	6.90	4.05	9.46

IR: Incidence rate.

Analysis of the risk maps indicates a mixed pattern of persistence and spatial displacement of HIV/AIDS hotspots over the four decades analyzed. The maintenance of high risk in the central region (Headquarters District) was observed since the 1980s, especially in neighborhoods historically linked to greater urban mobility, commerce, and nightlife. However, from the 2000s onwards, a progressive shift of risk towards the peripheral macro-areas is noted, particularly in the districts of Campo Grande (north), Ouro Verde (south), and Sousas/Joaquim Egídio (east).

The map showing the distribution of controls selected for the risk model analyses, during the study period for both notification criteria (AIDS and HIV), can be seen in Figure S1 (supplementary material).

## DISCUSSION

The spatial distribution of AIDS and HIV risk revealed an expansion into peripheral areas, some of which are characterized by high social vulnerability and poorer socioeconomic conditions. The phenomenon of "peripheralization," observed in this study, is highlighted in other works that trace the dynamics of the epidemic over time and the negative phenomena associated with it — specifically, both the spread of HIV into inland municipalities and its migration toward the outskirts of cities^5,15,16^. The negative impacts of the disease are a cause for concern; these impacts vary depending on factors such as human development levels, population distribution (which is not uniform across a city's total land area), population growth, and the concentration of people in peripheral urban zones^17^.

The 1980s were marked by a rise in AIDS risk, particularly in the metropolitan regions of São Paulo and Rio de Janeiro. This trend was characterized by a concentration of cases predominantly among men and individuals of high socioeconomic status — a status that, at the time, facilitated travel to other countries where cases had already been reported, thereby increasing the likelihood of contact with infected individuals. Other affected groups included homosexual and bisexual men, hemophiliacs and/or blood transfusion recipients, and injection drug users^18,19^. Some of these characteristics contributed to a heightened risk of the disease among residents in central Campinas, given that during the years in question this area served as the focal point for the city's population, housing its bohemian districts and tertiary sector activities^20^.

The District of Joaquim Egídio was a rural district of Campinas between 1980 and 1989, characterized as an urban enclave physically isolated from the central region; however, starting in the 1990s, spatial occupation within the district intensified, as did the expansion of leisure activities such as bars and restaurants^20^. The indications of risk appearing along the edges of the city map may be subject to an "edge effect," given that the number of control samples in this region was low, thereby amplifying the risk in the presence of cases^21^.

The intensity of population growth in the peripheral areas during the 1990s can be explained, in part, by increased intra-urban migration flows following intense real estate speculation, which led to a redistribution of Campinas's population toward more distant neighborhoods — as well as toward municipalities within the metropolitan region — in search of more affordable housing conditions and lifestyles^22^.

The persistent social crisis and income inequality observed during this period displaced the poorer segments of the population toward the peripheries, driving them out of central areas where social amenities and healthcare infrastructure were concentrated. A rise was recorded in the number of "cortiços" (tenements), "favelas" (slums), and clandestine or irregular subdivisions lacking basic habitability standards, thereby negatively impacting socio-environmental indicators^22,23^.

Since the 2000s, a continuous and expanding urban sprawl has been observed along the highways connecting Campinas to neighboring municipalities, with particular densification in the regions bordering Jaguariúna and Pedreira^24^. This expansion coincides with the growth of university and technology-related activities in these areas, driven by the presence of major higher education and research institutions such as Unicamp, the Pontifical Catholic University of Campinas (PUC-Campinas), the Campinas Faculty (Facamp), and the Telecommunications Research and Development Center (CPQD)^24^.

This transition can be interpreted as a reflection of multiple interconnected phenomena: (1) the process of the peripheralization of poverty and vulnerability, as described in socio-spatial analyses of the São Paulo metropolitan areas and Campinas^22,23^; (2) disorderly urban expansion and the formation of a continuous urban sprawl extending toward the municipality's outskirts—a phenomenon widely documented by local urban planning studies^23,24^; and (3) the increasing functional integration of the Campinas Metropolitan Region, characterized by migratory flows, daily mobility, and exchanges with neighboring municipalities such as Hortolândia, Pedreira, and Indaiatuba^24^. Thus, the territorial pattern of the epidemic came to reflect new population and health-related centralities, necessitating a territorial reorganization of prevention and surveillance efforts.

It is estimated that Campinas receives approximately 60,000 university students annually, hailing from various municipalities within the state of São Paulo, from other states, and even from abroad. This influx contributes to an intense dynamic of inter-regional mobility and exchange, including social, cultural, and sexual practices, that may influence the dissemination patterns of sexually transmitted infections (STIs)^25^. Previous studies demonstrate that university students constitute a potential risk group for HIV, owing both to the initiation or intensification of sexual activity and to lower condom use and reduced risk perception^26-28^. University-related migration flows, combined with temporary academic mobility (such as exchange programs, conferences, and short-term courses), may increase the number of interpersonal contacts and facilitate the spread of STIs, particularly in contexts characterized by low adherence to regular testing and inconsistent condom use^25-28^.

Analyzing growth trends since 2010, a predominance is observed among young adults aged 20 to 39, with a detection rate for reported HIV infection cases of 15.6 per 100,000 inhabitants^29^. Even with a trend toward declining risk intensity for AIDS and HIV in Campinas starting in 2010^30^, the city recorded an AIDS incidence rate of 11.6 per 100,000 inhabitants in 2015, alongside an exponential increase in the detection and reporting of new HIV cases^31^.

The phenomenon of rising case numbers among individuals aged 60 and older has been described in studies conducted in the State of São Paulo, which point to a trend of increasing HIV/AIDS mortality among older individuals between 2010 and 2020, particularly among men. Contributing factors include the aging of the sexually active population, the absence of preventive policies tailored to older people, the use of erectile dysfunction medications, and a low perception of risk among health care professionals. The persistence of risk in this age group underscores the importance of specific educational initiatives and active testing for older adults who remain sexually active^32-34^. Although they represent a smaller absolute proportion of cases, older persons constitute a neglected population within prevention programs. The data indicate an emerging epidemiological pattern of late-onset infection, frequently diagnosed at advanced stages of the disease, thereby elevating the risk of complications and mortality^32-34^.

As a limitation, it is noteworthy that HIV data predating the national mandatory reporting system, which was instituted only in 2014, are more susceptible to underreporting and data entry inconsistencies; this may affect temporal comparability and the identification of more recent patterns within the epidemic. The inability to geocode records with incomplete addresses may also have reduced the sensitivity of the spatial analysis in areas of higher social vulnerability. Furthermore, the migration of cases originating from neighboring municipalities, given Campinas's role as a regional referral hub, may have contributed to an overestimation of risk in specific regions of the municipality. Nevertheless, the identified territorial patterns remained consistent across the decades analyzed, thereby reinforcing the robustness of the geospatial estimates produced.

An important limitation regarding the quality and completeness of HIV data prior to 2014 must be taken into account. Although the state of São Paulo had already adopted recommended HIV reporting practices as early as the 2000s, mandatory reporting at the national level was officially instituted only through Ministry of Health Ordinance No. 1,271, dated June 6, 2014^1^. This regulatory framework officially established the mandatory reporting of HIV infection cases to the SINAN system, extending this requirement to all public and private health services.

Consequently, records predating 2014 exhibit a greater susceptibility to underreporting, inconsistencies, and a lack of standardization in data collection and entry, in addition to a potential reliance on the local initiative of state and municipal health departments. This implies heterogeneity in the coverage and timeliness of records, which may affect time-series analysis and spatial modeling, particularly regarding early detection and more recent territorial patterns of transmission. Thus, findings related to HIV should be interpreted with caution, especially for the decades preceding the legal mandate for notification. Nevertheless, geospatial analysis enables the identification of consistent patterns of concentration and territorial expansion of the infection, reinforcing the need to strengthen active surveillance and the timely detection of cases in high-risk territories.

It is concluded that the spatial distribution of HIV infection risk and AIDS notifications in Campinas exhibits heterogeneity across the different periods of the epidemic. High-risk areas overlapped across the decades; however, certain peripheral areas and the central region were consistently identified as high-risk zones throughout the decades under study. Data georeferencing aids health surveillance, proving to be a powerful tool for monitoring the spatial distribution of disease risks, serving as a starting point for priority prevention and care initiatives.

These findings underscore the importance of incorporating geospatial surveillance as a supporting tool for territorial health management, particularly within the scope of primary care and combined prevention policies.
