# Supplementary material for: Spatial distribution of HIV/AIDS risk in Campinas-SP (1980–2019): a geospatial analysis by decade
Source: Rev Bras Epidemiol. 2026 May 8;29:e260021. doi: 10.1590/1980-549720260021 (PMC13159491; doi:10.1590/1980-549720260021)

**Figure S1 (Supplementary material).** Spatial distribution of controls following reliability determination, compared to the distribution of cases during the study period. Campinas, SP, 1980 to 2019.


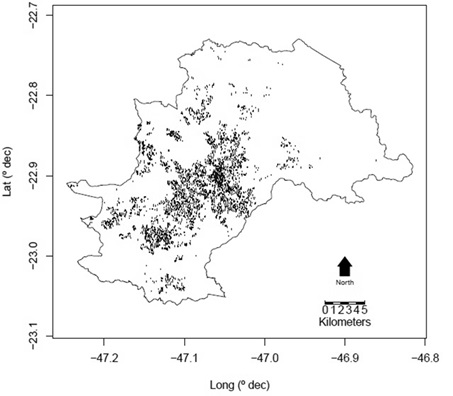

Supplement: SUPPLEMENTARY MATERIAL [file 1980-5497-rbepid-29-e260021-suppl1.docx]
