# Supplementary material for: Spatial distribution of HIV/AIDS risk in Campinas-SP (1980–2019): a geospatial analysis by decade
Source: Rev Bras Epidemiol. 2026 May 8;29:e260021. doi: 10.1590/1980-549720260021 (PMC13159491; doi:10.1590/1980-549720260021)
Supplement: Translation

# Translation: Distribuição espacial do risco de HIV/AIDS em Campinas-SP (1980-2019):uma análise geoespacial por década

**DOI:** 10.1590/1980-549720260021.2

**Authors:** Márcio Cristiano de Melo, Celso Stephan, Ricardo Carlos Cordeiro, Valeria Correia de Almeida, Maria Rita Donalisio

**Affiliations:** I Faculdade de Medicina São Leopoldo Mandic – Araras (SP), Brasil.; II Universidade Estadual de Campinas, Departamento de Saúde Coletiva – Campinas (SP), Brasil.; III Secretaria Municipal de Saúde de Campinas, Departamento de Vigilância em Saúde – Campinas (SP), Brasil.

**Keywords:** HIV, AIDS, Análise espacial, Vigilância em saúde pública, Fatores socioeconômicos, Epidemiologia

## Abstract

**Objetivo:**

Analisar a distribuição espacial do risco de casos de AIDS (1980–2019) e infecção por vírus da imunodeficiência humana — HIV (2000–2019) no município de Campinas.

**Métodos:**

Estudo caso-controle geoespacial com dados do Sistema de Informação de Agravos de Notificação. Foram incluídos todos os casos de AIDS e HIV em indivíduos com 13 anos ou mais, excluindo gestantes e notificações por óbito. A geocodificação foi realizada com o uso da base cartográfica oficial do município, utilizando coordenadas UTM (SAD69). A modelagem do risco espacial foi conduzida por meio de modelos aditivos generalizados (GAM), com aplicação do método de Kelsall e Diggle, utilizando pares de coordenadas geográficas como preditores não paramétricos. Os resultados foram estratificados por década e comparados entre períodos.

**Resultados:**

Foram incluídos 11.372 casos de AIDS (1980–2019) e 3.329 casos de HIV (2000–2019). Observou-se padrão persistente de risco elevado no Distrito Sede desde os anos 1980, com deslocamento progressivo para distritos periféricos (Campo Grande, Ouro Verde, Sousas e Joaquim Egídio). A periferização dos casos de AIDS e da infecção pelo HIV coincidiu com processos de urbanização acelerada e desigualdade socioespacial. As maiores taxas de incidência de HIV e AIDS ocorreram entre adultos de 20 a 39 anos, com crescimento relevante entre idosos. A análise demonstrou sobreposição parcial de hotspots e expansão de áreas de risco.

**Conclusão:**

A distribuição espacial de HIV/AIDS em Campinas é heterogênea e dinâmica, marcada por persistência em áreas centrais e expansão para regiões periféricas vulneráveis. O georreferenciamento mostrou-se ferramenta estratégica para orientar ações de prevenção e controle territorialmente sensíveis.

## INTRODUÇÃO

A disseminação da infecção por HIV ao longo do tempo afetou as principais metrópoles do Brasil, avançando, em seguida, para municípios de médio e de pequeno porte. Além da variabilidade geográfica no acesso a diagnóstico e tratamento, observa-se concentração da epidemia em populações-chave, conforme apontam os dados do Ministério da Saúde^1,2^.

A maior detecção de casos em capitais e centros metropolitanos tem sido apontada desde o início da epidemia^3^. Esses centros possuem as maiores densidades demográficas e maior interação com cidades menores, levando-se em consideração a facilidade de deslocamentos, presença de maiores oportunidades de emprego, negócios, além de maior oferta de equipamentos sociais e urbanos^4,5^.

O estado de São Paulo caracterizou-se pelo aumento progressivo da detecção de casos de AIDS na maioria dos municípios ao longo da série histórica da doença. Observou-se crescimento *in situ*, isto é, no mesmo local e independentemente de variáveis sociodemográficas. O risco de infecção aumentou e espalhou-se pelo estado a partir dos anos 2000, evidenciando o processo de interiorização da epidemia^6^.

A análise da distribuição espacial da infecção permite identificar áreas de maior concentração relativa de risco, possibilitando a priorização territorial de intervenções. Assim, a abordagem de risco espacial contribui para superar as informações sobre a distribuição espacial dos casos, pois pondera as notificações levando em conta a população sob risco^7^.

A distribuição espacial da epidemia apresenta diferentes padrões, caracterizados pela maior concentração de casos em bairros com piores condições socioeconômicas, nos quais predominam casos entre homens. Outro padrão verificado foi o de maior suscetibilidade à infecção em mulheres em bairros periféricos, nos quais a relação entre a infecção em homem/mulher é menor. Tais padrões territoriais facilitam a abordagem de populações vulneráveis por parte de setores de saúde^8^.

O objetivo deste estudo foi analisar a distribuição espacial do risco de ocorrência dos casos de AIDS (notificação) durante quatro décadas (1980 a 2017) e do HIV a partir dos anos 2000, no município de Campinas.

## MÉTODOS

Trata-se de um estudo caso-controle geoespacial sobre o risco espacial de AIDS e do HIV da totalidade de casos de ambas as categorias de notificação em maiores de 13 anos no município de Campinas/SP. Os casos foram registrados pelo Departamento de Vigilância em Saúde (DEVISA) da Secretaria Municipal de Saúde de Campinas no período de 1980 a 2019. Após revisão do banco de dados, foram excluídos casos em gestantes, por conta do fluxo diferenciado de testagem no pré-natal, que poderia superestimar o risco em áreas com maior cobertura assistencial, e notificações cujo critério diagnóstico foi o óbito, pois esses registros apresentam endereços ausentes ou imprecisos, inviabilizando a geocodificação e podendo introduzir erros espaciais; reconhece-se que tal exclusão pode subestimar o risco em áreas de maior vulnerabilidade.

Campinas é considerada uma cidade polo de desenvolvimento industrial de alta tecnologia e possui índice de desenvolvimento humano de 0,805, ocupando, no *ranking* das cidades brasileiras, a posição de número 28. A população estimada de Campinas em 2018 era de 1.194.094 habitantes^9^. O município assemelha-se ao padrão geral das grandes cidades brasileiras em aspectos epidemiológicos e de controle de doenças transmissíveis. A cidade é um centro de referência regional médico-hospitalar e apresentou taxa de detecção de 17,6 casos de aids por 100 mil habitantes no ano de 2017^10^, possui um programa estruturado de prevenção e assistência aos pacientes com HIV/AIDS desde o início da epidemia.

A variável endereço foi identificada na ficha de notificação epidemiológica do Sistema de Informação de Agravos de Notificação (SINAN) para a inclusão no cálculo do risco espacial dos casos. Utilizou-se a base cartográfica elaborada pela Sociedade de Abastecimento de Água e Saneamento de Campinas em 2000 com o *software* AutoCAD, atualizada periodicamente pela Secretaria Municipal de Planejamento e Secretaria Municipal de Saúde (última atualização realizada em 2014). Por meio do *software* ArcView foram incluídos novos bairros, locais de invasão, instalações urbanas e conjuntos habitacionais, além de atualização de dados de interesse da área da saúde. Para a elaboração dessa base cartográfica foram utilizados os seguintes parâmetros: Elipsoide Internacional de Hayfor, Projeção Universal Transversa de Mercator, Datum SAD69 e limite do Município do Instituto Brasileiro de Geografia e Estatística (IBGE) — ajustado.

A localização dos casos na base cartográfica foi feita por meio dos endereços de moradia pelo método de geocodificação do *software* ArcMap 10.5, após correção e padronização dos dados na base de arruamento do município. Foram excluídos endereços que não permitiam a exata localização do ponto no mapa, como resultado de insuficiência da informação ou inexatidão do nome do logradouro, população carcerária (por ausência de correspondência com endereços civis válidos), abrigos e instituições de permanência de pessoas que vivem com HIV e AIDS. Uma vez geocodificados, os casos localizados na base tiveram as suas coordenadas geográficas anexadas em UTM SAD69.

Utilizou-se o método desenvolvido por Kelsall e Diggle^11^, no qual o risco espacial é estimado por meio do ajuste de um modelo aditivo generalizado (GAM), incluindo a verificação de áreas de significância estatística. A escolha do modelo aditivo generalizado (GAM) para estimar o risco espacial de HIV/AIDS neste estudo justifica-se por sua flexibilidade na modelagem de relações não lineares entre a variável resposta e coordenadas geográficas, sem a necessidade de especificar uma estrutura paramétrica rígida^12^. Isso é particularmente adequado para modelar padrões de risco espacialmente contínuos e suavizados, especialmente quando se trabalha com dados pontuais georreferenciados.

Foi ajustado para cada período um modelo utilizando o *status* diagnóstico de AIDS e sorologia positiva para HIV como variável resposta (casos = 1 e controles = 0). Os controles foram obtidos por meio do pacote AmostraBrasil sob Licença Geral Pública (GPL), desenvolvido para fins de análises espaciais de dados epidemiológicos^13^. Como variável preditora não paramétrica, utilizou-se o par de coordenadas geográficas referentes à residência de casos e controles^7^. Desse modo, os valores preditos pelo modelo representam a estimativa de distribuição do risco de AIDS (décadas de 1980, 1990, 2000 e 2010 até 2019) e de HIV (década de 2000 e 2010 a 2019). Este período foi escolhido em razão da recomendação estadual da notificação do HIV em 2000, tornada compulsória nacionalmente apenas em 2014^14^.

Os modelos foram calculados no *software* R, versão 3.4.1, utilizando-se a função GAM do *package mgcv*. Os números nas linhas no mapa indicam quantas vezes o risco é maior naquela região, comparada com o valor de referência 1, que é o risco médio do território.

Para o cálculo dos coeficientes de incidência, utilizou-se o número de casos notificados em Campinas de HIV e AIDS de acordo com as faixas etárias (13 a 19, 20 a 29, 30 a 39, 40 a 49, 50 a 59 e 60 anos ou mais) como numerador e a população específica por faixa etária como denominador, entre os anos 2010 e 2019.

O projeto foi aprovado pelo Comitê de Ética de Pesquisas em Seres Humanos — FCM/Unicamp sob Parecer nº 1.776.974.

### Declaração de Disponibilidade de Dados:

Todo o conjunto de dados que dá suporte aos resultados deste estudo está disponível mediante solicitação à Secretaria Municipal de Saúde de Campinas, por meio do Departamento de Ensino, Pesquisa e Saúde Digital (DEPS). O conjunto de dados não está publicamente disponível por conter informações que podem comprometer a privacidade dos participantes da pesquisa.

## RESULTADOS

Foram incluídos na análise 11.372 casos de AIDS diagnosticados entre os anos de 1980 e 2019, e 3.329 casos de HIV diagnosticados no período de 2000 a 2019. Destes, utilizou-se a localização de 100% dos registros dos casos a respeito das coordenadas de latitude e longitude na base cartográfica.

Os mapas na Figura 1 mostram os modelos lineares generalizados da distribuição espacial do risco de AIDS nos quatro períodos estudados. Ao longo do tempo, observam-se diferenças de risco de AIDS em moradores no território da cidade. A cor vermelha indica os locais de Campinas onde o risco da doença foi significativamente maior que a média no período específico. Na década de 1980 a 1989, o maior risco de AIDS foi evidenciado em moradores dos bairros do centro da cidade, em regiões periféricas com condições precárias de vida, na Macroárea Sul (em bairros próximos do aeroporto de Viracopos e vizinhos à cidade de Indaiatuba) e na região do Distrito de Joaquim Egídio.

**Figura 1:** Distribuição espacial do risco de AIDS após ajuste de modelo aditivo generalizado nos quatro períodos do estudo. Campinas, SP, 1980 a 2019.

Na década de 1990, o maior risco ainda era observado no Distrito Sede e Macroárea Sul, com expansão para a Macroárea Norte da cidade, próximos do Complexo Penitenciário de Hortolândia, município que faz fronteira com Campinas. Entre os anos de 2000 e 2009, a análise indica manutenção do risco elevado no Distrito Sede do município, Macroárea Norte, divisa com Hortolândia, e aponta aumento significativo do risco no Distrito de Sousas. O mapa que apresenta os dados referentes aos anos de 2010 a 2019 indica risco maior de AIDS no Distrito de Joaquim Egídio e Distrito de Sousas, Distrito Sede e na Macroárea Sul (Figura 1).

A Figura 2 destaca os modelos lineares generalizados da distribuição espacial do risco de HIV entre os anos de 2000 e 2019. Evidencia-se na primeira década desse período (2000 a 2009) um risco elevado em cinco vezes em bairros localizados na Macroárea Norte de Campinas pertencentes às Macroárea Norte e Distrito de Joaquim Egídio. No período de 2010 a 2019, o maior risco, cerca de 1,5 vez maior, é observado também na Macroárea Leste-Nordeste, Distrito de Sousas, e com a mesma magnitude em bairros do Distrito Sede e na Macroárea Sul do município, divisa com Indaiatuba. Todos os municípios citados que fazem fronteira com Campinas pertencem à região metropolitana de Campinas.

**Figura 2:** Distribuição espacial do risco de vírus da imunodeficiência humana (HIV) após ajuste de modelo aditivo generalizado nos dois períodos do estudo. Campinas, SP, 2000 a 2019.

De acordo com a Tabela 1, constata-se tendência crescente dos índices referentes aos casos de HIV e decrescentes com os casos de AIDS. Os maiores coeficientes de HIV e AIDS são referidos entre as faixas etárias compreendidas entre os 20 e os 39 anos, com registro em 2017: entre adultos de 20 a 29 e 30 a 39 anos, 39,0 e 33,2 novos casos de HIV por 100 mil habitantes e 19,9 e 29,2 novos casos de AIDS por 100 mil habitantes, respectivamente. Um achado significativo diz respeito à manutenção de taxas significativas de HIV/AIDS entre pessoas idosas, especialmente na faixa etária acima de 60 anos. A Tabela 1 demonstra que, mesmo com coeficientes absolutos mais baixos, a taxa de incidência de HIV em idosos quase triplicou entre 2010 (2,2/100 mil) e 2017 (5,5/100 mil).

**Tabela 1:** Coeficiente de Incidência de vírus da imunodeficiência humana (HIV) e AIDS por 100 mil habitantes (população específica) de acordo com faixas etárias em anos. Campinas, 2010 a 2019.

		13–19	20–29	30–39	40–49	50–59	≥ 60	Total
2010	CI HIV	1,73	35,77	32,13	17,40	13,73	2,21	19,72
	CI AIDS	0,86	37,73	55,55	45,12	23,43	11,79	32,13
2011	CI HIV	6,06	30,21	37,31	12,76	6,30	2,83	18,28
	CI AIDS	6,92	39,13	52,77	49,78	29,16	12,02	34,19
2012	CI HIV	5,18	36,72	28,16	26,56	16,17	3,39	21,34
	CI AIDS	3,45	39,23	49,55	35,42	23,10	8,13	29,20
2013	CI HIV	5,17	45,06	41,36	19,44	9,79	4,55	23,71
	CI AIDS	6,89	30,72	40,85	38,87	30,14	8,45	27,59
2014	CI HIV	5,17	58,41	43,57	18,01	19,95	3,74	27,68
	CI AIDS	3,44	29,20	35,55	34,77	19,95	3,12	22,71
2015	CI HIV	10,40	74,76	46,84	23,34	17,42	4,78	32,62
	CI AIDS	3,47	30,75	29,09	29,48	15,24	8,36	20,93
2016	CI HIV	9,63	82,08	59,15	30,82	18,59	6,30	37,86
	CI AIDS	4,38	28,97	28,84	24,78	22,88	9,16	21,01
2017	CI HIV	6,20	46,09	32,56	23,76	19,76	5,49	23,81
	CI AIDS	3,54	33,07	27,70	26,14	21,87	7,14	21,10
2018	CI HIV	13,45	115,50	82,38	43,75	30,68	7,37	52,62
	CI AIDS	0,90	23,54	22,29	21,00	11,16	4,74	15,02
2019	CI HIV	4,54	38,68	27,13	14,31	10,35	2,02	17,24
	CI AIDS	3,63	12,16	16,96	9,73	6,90	4,05	9,46

CI: Coeficiente de Incidência.

A análise dos mapas de risco indica um padrão misto de persistência e deslocamento espacial de *hotspots* de HIV/AIDS ao longo das quatro décadas analisadas. Observou-se a manutenção do risco elevado na região central (Distrito Sede) desde os anos 1980, especialmente em bairros historicamente vinculados à maior mobilidade urbana, ao comércio e à vida noturna. Contudo, a partir dos anos 2000, nota-se um deslocamento progressivo do risco em direção às macroáreas periféricas, com destaque para os distritos de Campo Grande (norte), Ouro Verde (sul) e Sousas/Joaquim Egídio (leste).

O mapa com a distribuição dos controles selecionados para as análises do modelo de risco, no período de estudo para ambos os critérios de notificação (AIDS e HIV), pode ser visto na Figura S1 (material suplementar).

## DISCUSSÃO

A distribuição espacial do risco de AIDS e HIV mostrou uma expansão em áreas periféricas, algumas destas com alta vulnerabilidade social e piores condições socioeconômicas. O fenômeno da periferização, observado neste estudo, é salientado em trabalhos que apontam a dinâmica da epidemia ao longo do tempo e os fenômenos negativos relacionados a ela, tanto a interiorização do HIV pelos municípios quanto a migração para as periferias das cidades^5,15,16^. Tornam-se preocupantes os impactos negativos da doença, que variam em razão do desenvolvimento humano, da distribuição da população, que não é uniforme em relação à extensão territorial das cidades, do crescimento populacional e da concentração populacional em áreas urbanas periféricas^17^.

A década de 1980 ficou marcada pelo aumento do risco de AIDS, principalmente em regiões metropolitanas de São Paulo e Rio de Janeiro, caracterizado pela concentração dos casos majoritariamente em homens e indivíduos com alto nível socioeconômico — o que possibilitava, na época, viagens para outros países que já possuíam casos notificados e possíveis contatos com pessoas já doentes, homo/bissexuais, portadores de hemofilia e ou receptores de sangue e usuários de drogas injetáveis (UDI)^18,19^. Algumas dessas características favoreceram o maior risco da doença em moradores no centro de Campinas, uma vez que, nos anos em questão, era lá que se concentrava a população da cidade, em zonas boêmias e atividades terciárias^20^.

O Distrito de Joaquim Egídio era um distrito rural de Campinas entre os anos de 1980 e 1989, caracterizado como um núcleo urbano fisicamente isolado da região central, porém a partir da década de 1990 a ocupação espacial do distrito se intensificou, como também se ampliaram as atividades de lazer, como bares e restaurantes^20^. As imagens que indicam risco nas bordas do mapa da cidade podem estar sujeitas ao efeito de borda, uma vez que o número de controles foi baixo nessa região, ampliando o risco na presença de casos^21^.

A intensidade do crescimento da população nas periferias na década de 1990 pode ser explicado, em parte, pelo aumento dos fluxos migratórios intraurbanos após intensa especulação imobiliária, com redistribuição da população campineira para bairros mais distantes e também para os municípios pertencentes à região metropolitana, em busca de condições mais econômicas de moradia e estilo de vida^22^.

A constante crise social e a desigualdade de renda observadas nesse período expulsaram a população mais pobre para as periferias, vinda das áreas centrais onde há equipamentos sociais e infraestrutura de atenção à saúde. Registrou-se aumento de cortiços, favelas e loteamentos clandestinos e irregulares que não possuíam condições de habitabilidade, interferindo nos indicadores socioambientais^22,23^.

A partir dos anos 2000, observa-se uma mancha urbana contínua e crescente ao longo das rodovias que conectam Campinas a municípios vizinhos, com adensamento especialmente nas regiões limítrofes a Jaguariúna e Pedreira^24^. Essa expansão coincide com o crescimento das atividades universitárias e tecnológicas nessas áreas, impulsionado pela presença de importantes instituições de ensino superior e pesquisa, como a Unicamp, Pontifícia Universidade Católica de Campinas (PUC-Campinas), Faculdade de Campinas (Facamp) e Centro de Pesquisa e Desenvolvimento em Telecomunicações (CPQD)^24^.

Essa transição pode ser interpretada como um reflexo de múltiplos fenômenos interligados: (1) o processo de periferização da pobreza e da vulnerabilidade, descrito em análises socioespaciais das metrópoles paulistas e de Campinas^22,23^; (2) a expansão urbana desordenada e a formação de uma mancha urbana contínua em direção às bordas do município, fenômeno amplamente documentado por estudos urbanísticos locais^23,24^; e (3) a crescente integração funcional da Região Metropolitana de Campinas, marcada por fluxos migratórios, mobilidade cotidiana e intercâmbios com municípios vizinhos como Hortolândia, Pedreira e Indaiatuba^24^. Assim, o padrão territorial da epidemia passou a refletir novas centralidades populacionais e sanitárias, exigindo uma reorganização territorial das ações de prevenção e vigilância.

Estima-se que Campinas receba cerca de 60 mil estudantes universitários por ano, oriundos de diversos municípios paulistas, de outros estados e até do exterior. Esse fluxo contribui para uma dinâmica intensa de mobilidade e intercâmbio inter-regional, incluindo práticas sociais, culturais e sexuais que podem influenciar o padrão de disseminação de infecções sexualmente transmissíveis (IST)^25^. Estudos prévios demonstram que estudantes universitários representam um grupo de risco potencial para o HIV, tanto pelo início ou intensificação da vida sexual quanto pelo menor uso de preservativos e percepção de risco reduzida^26-28^. O fluxo migratório universitário, somado à circulação acadêmica temporária (intercâmbios, congressos e cursos de curta duração), pode ampliar o número de contatos interpessoais e facilitar a propagação de IST, especialmente em contextos de baixa adesão à testagem regular e uso inconsistente de preservativos^25-28^.

Analisando as tendências de crescimento a partir de 2010, observa-se predominância entre jovens e adultos de 20 a 39 anos, com taxa de detecção de casos notificados de infecção pelo HIV de 15,6 por 100 habitantes^29^. Mesmo com a tendência de declínio na intensidade de risco de AIDS e HIV em Campinas a partir do ano de 2010^30^, a cidade registrou em 2015 uma taxa de incidência de AIDS de 11,6 por 100 mil habitantes^29^ e aumento exponencial de detecção e notificação de novos casos de HIV^31^.

O fenômeno de aumento dos casos em pessoas com 60 anos ou mais tem sido descrito em estudos realizados no Estado de São Paulo, que apontam tendência de crescimento da mortalidade por HIV/AIDS entre idosos entre 2010 e 2020, especialmente entre homens. Entre os fatores contribuintes, destacam-se o envelhecimento da população sexualmente ativa, a ausência de políticas preventivas voltadas ao público idoso, o uso de medicamentos para disfunção erétil e a baixa percepção de risco por parte dos profissionais de saúde. A persistência de risco nessa faixa etária reforça a importância de ações educativas específicas e de testagem ativa no envelhecimento com sexualidade ativa^32-34^. Embora representem menor proporção absoluta, os idosos constituem uma população negligenciada nos programas de prevenção. Os dados indicam um padrão epidemiológico emergente de infecção tardia, frequentemente com diagnóstico em estágios avançados da doença, elevando o risco de complicações e mortalidade^32-34^.

Como limitação, destaca-se que os dados de HIV anteriores à notificação compulsória nacional, instituída apenas em 2014, apresentam maior suscetibilidade à subnotificação e inconsistências de preenchimento, o que pode afetar a comparabilidade temporal e a identificação de padrões mais recentes da epidemia. A impossibilidade de geocodificar registros com endereços incompletos também pode ter reduzido a sensibilidade da análise espacial em áreas de maior vulnerabilidade social. Além disso, a migração de casos oriundos de municípios vizinhos, dada a função de referência regional de Campinas, pode ter contribuído para a superestimação do risco em regiões específicas do município. Ainda assim, os padrões territoriais identificados mantiveram consistência entre as décadas analisadas, reforçando a robustez das estimativas geoespaciais produzidas.

Deve-se considerar uma importante limitação relacionada à qualidade e à completude dos dados de HIV anteriores ao ano de 2014. Embora o estado de São Paulo tenha adotado a notificação do HIV de forma recomendada já a partir dos anos 2000, a notificação compulsória em nível nacional somente foi instituída pela Portaria nº 1.271, de 6 de junho de 2014^1^, do Ministério da Saúde. Esse marco normativo estabeleceu oficialmente a obrigatoriedade da notificação dos casos de infecção pelo HIV no SINAN, estendendo a exigência a todos os serviços de saúde públicos e privados.

Como consequência, os registros anteriores a 2014 apresentam maior suscetibilidade à subnotificação, inconsistências e ausência de padronização na coleta e inserção dos dados, além da possível dependência da iniciativa local das secretarias estaduais e municipais de saúde. Isso implica heterogeneidade na cobertura e oportunidade dos registros, o que pode afetar a análise de séries temporais e a modelagem espacial, especialmente no que se refere à detecção precoce e aos padrões territoriais mais recentes de transmissão. Assim, os achados relativos ao HIV devem ser interpretados com cautela, especialmente nas décadas anteriores à obrigatoriedade legal da notificação. Ainda assim, a análise geoespacial permite a identificação de padrões consistentes de concentração e expansão territorial da infecção, reforçando a necessidade de fortalecimento da vigilância ativa e da captação oportuna de casos nos territórios de maior risco.

Conclui-se que a distribuição espacial do risco de infecção por HIV e notificação de AIDS apresenta heterogeneidade em Campinas nos diferentes períodos da epidemia. As áreas de maior risco sobrepuseram-se nas décadas, entretanto algumas áreas periféricas e a região central foram identificadas como de maior risco no decorrer das décadas estudadas. O georreferenciamento de dados auxilia na vigilância em saúde, mostrando ser um poderoso instrumento para acompanhar a distribuição de riscos de doenças no território, ponto de partida para ações prioritárias de prevenção e assistência.

Os achados reforçam a importância de incorporar a vigilância geoespacial como ferramenta de apoio à gestão territorial da saúde, sobretudo no âmbito da atenção primária e das políticas de prevenção combinada.

## MATERIAL SUPLEMENTAR

MATERIAL SUPLEMENTAR
